# HadS, a membrane-associated 2-haloacid dehalogenase from the moderate halophile *Halobacillus andaensis* NEAU-ST10-40^T^, enhances saline-alkaline tolerance when heterologously expressed in *Escherichia coli* KNabc

**DOI:** 10.3389/fmicb.2026.1823317

**Published:** 2026-06-08

**Authors:** Li Shao, Yunhan Xu, Changjiang He, Shihan Zhao, Yaohua Li, Juquan Jiang

**Affiliations:** Department of Microbiology and Biotechnology, College of Life Sciences, Northeast Agricultural University, Harbin, China

**Keywords:** 2-haloacid dehalogenases, HadS, *Halobacillus andaensis*, moderate halophile, saline-alkaline tolerance

## Abstract

**Introduction:**

2-Haloacid dehalogenases are a subgroup of the haloacid dehalogenase superfamily and catalyze the hydrolytic dehalogenation of 2-haloalkanoic acids. To date, few HAD superfamily members have been reported to be potentially associated with saline-alkaline tolerance.

**Methods:**

In this study, a HAD superfamily protein, HadS, consisting of 220 amino acid residues, was identified from the moderately halophilic bacterium *Halobacillus andaensis* NEAU-ST10-40^T^ through genomic library screening and functional complementation in the Na^+^/H^+^ antiporter-deficient *Escherichia coli* KNabc strain. Bioinformatic prediction, fluorescence quenching recovery assays, Western blotting, fluorescence spectroscopy, and atomic absorption spectroscopy were used to investigate its characteristics and biological functions.

**Results:**

No transmembrane domain was predicted in HadS, and no obvious Na^+^(Li^+^, K^+^)/H^+^ antiport activity was detected, indicating that HadS does not function as a typical cation/proton antiporter. Western blotting detected HadS in membrane-enriched fractions, suggesting a possible membrane-associated distribution. Fluorescence spectroscopy provided preliminary evidence of potential 2-haloacid dehalogenase activity. In addition, heterologous expression of HadS significantly reduced intracellular Na+ accumulation and enhanced the saline-alkaline tolerance of recombinant *E. coli* KNabc.

## 1 Introduction

The haloacid dehalogenase-like hydrolase superfamily is one of the largest enzyme superfamilies found across all domains of life, characterized by a conserved *α*/*β* Rossmann-like fold and a distinctive catalytic motif that enables its members to act on a remarkably broad spectrum of substrates (Leader and Milner-White, 2023; Pathira Kankanamge et al., 2023; Ling et al., 2021; Avs and Balaram, 2025). Although originally named after the haloacid dehalogenases (HADs), the superfamily is predominantly composed of phosphoesterases, ATPases, phosphonatases, and sugar phosphomutases, each if which perform distinct physiological functions in cellular metabolism, including phosphorus homeostasis, ion transport, signal transduction, and xenobiotic compound degradation (Grigorian et al., 2021; Grigorian et al., 2021; Wang et al., 2021; Kaur et al., 2024). Among the HAD subgroups, 2-haloacid dehalogenases are particularly important due to their ability to catalyze the hydrolytic dehalogenation of 2-haloalkanoic acids, which have significant applications in the fields of environmental protection and chemical synthesis (Wahhab et al., 2024), thereby attracting extensive attention in microbiology, biochemistry, and environmental science. Based on substrate stereoselectivity and product configuration, 2-Haloacid dehalogenase can be divided into four categories: L-2 haloacid dehalogenase (L-DEX, EC3.8.1.2), D-2-haloacid dehalogenase (D-DEX, EC3.8.1.9), DL-2 haloacid dehalogenase (configuration-inverting, EC3.8.1.10), and DL-2-haloacid dehalogenase (configuration-retaining, DL-DEXr, EC 3.8.1.11) (Avs and Balaram, 2025; Wang et al., 2021). As a member of the HAD superfamily, L-2-haloacid dehalogenase (L-2-HAD) catalyzes the dehalogenation of L-2-halogenated acids such as S-2-chloropropionic acid. L-2-HAD is widely distributed among various microorganisms in nature and remains the most extensively studied enzyme among 2-haloacid dehalogenases (Grigorian et al., 2021; Wang et al., 2021).

Halophiles have been widely recognized to harbor a larger number of Na^+^/H^+^ antiporters to cope with the stress of high saline–alkaline environment (Waditee-Sirisattha and Kageyama, 2024; Xing et al., 2024; Haja and Adams, 2021; Tsujii et al., 2020; Mesbah et al., 2009; Wang et al., 2023; Xamxidin et al., 2025; Dong et al., 2017; Shao et al., 2018; Shao et al., 2021). Strain NEAU-ST10-40^T^ was isolated from a Na_2_CO_3_-type saline-alkaline soil and identified as a novel species of the genus *Halobacillus*, designated *Halobacillus andaensis* (Wang et al., 2015). This strain is a moderate halophile capable of growing in 0.5–2.5 M NaCl, with an optimum at 1.4 M NaCl (Wang et al., 2015). This study speculates that halophiles such as NEAU-ST10-40^T^ strain may be forced to increase the number of Na^+^/H^+^ antiporters as an evolutionary strategy, potentially by modifying additional transporter families to function as Na^+^/H^+^ antiporters. These transporter families may include proteins that have not yet been functionally reported or proteins that possess known functions other than Na^+^/H^+^ antiporters. Evidence supporting this concept has been increasingly provided in the previous reports (Dong et al., 2017; Shao et al., 2018; Shao et al., 2021). Together, these findings imply that strain NEAU-ST10-40^T^ may have evolved a large repertoire of saline–alkaline tolerant genes, enabling survival and growth under prolonged saline–alkaline stress and tolerating NaCl concentrations up to 2.5 M.

Previous studies have shown that many saline–alkaline tolerance-related genes encode membrane transporters. In earlier screenings for saline–alkaline tolerance-related genes, several novel Na^+^/H^+^ antiporter genes, such as *upf0118*, *rdd*, and *nhaM*, were identified from strain NEAU-ST10-40^T^(Dong et al., 2017; Shao et al., 2018; Shao et al., 2021). These genes were predicted to encode proteins not previously reported to possess Na^+^/H^+^ antiport activity. This study first reports the functional analysis of a HAD superfamily member, designated HadS, composed of 220 amino acid residues from *H. andaensis* NEAU-ST10-40^T^. More importantly, the heterologous expression of HadS confers saline–alkaline tolerance in *Escherichia coli* KNabc. Combining bioinformatics analysis with functional analysis, this study propose that HadS functions as a membrane-associated 2-haloacid dehalogenase and extrudes intracellular Na^+^ across the cytoplasmic membrane coupled with the influx of extracellular proton during the haloacid dehalogenation reaction catalyzed using HadS. This mechanistic model explains why HadS may exhibit very low Na^+^(Li^+^, K^+^)/H^+^ antiport activity. This study also provides preliminary evidence that HadS, a HAD superfamily member, exhibits both 2-haloacid dehalogenase activity and the capacity to enhance saline-alkaline tolerance in *E. coli* KNabc.

## 2 Materials and methods

### 2.1 Strains, plasmids, and growth conditions

Strains and plasmids used in this study are listed in Table 1. *H. andaensis* NEAU-ST10-40^T^ was grown in 8% (w/v, optimum) NaCl modified S-G liquid medium at pH 8.0 containing 1.0% tryptone, 0.5% yeast extract, 0.5% casein, 0.2% KCl, 0.3% sodium citrate, 2.0% MgSO_4_·7H_2_O, and 8.0% NaCl (Wang et al., 2015). The major Na^+^/H^+^ antiporter-deficient *E. coli* mutant KNabc strains (such as *nhaA*: Km^R^, *nhaB*: Em^R^, and *chaA*: Cm^R^) were cultured in a KCl-modified Luria-Bertani (LBK) medium containing 1.0% tryptone, 0.5% yeast extract, and 87 mM KCl. Ampicillin was added to a final concentration of 50 μg/mL for the selection and growth of cells containing plasmids. For the salt tolerance test, 1% pre-cultures were inoculated into fresh LBK medium at pH 7.0, supplemented with NaCl or LiCl and then incubated at 37 °C. To test the effect of pH on cell growth, 1% pre-cultures were inoculated into fresh LBK medium with or without the addition of 50 mM NaCl at the indicated pH values, adjusted by adding the Hepes-Tris buffer to a final concentration of 100 mM, followed by incubation at 37 °C, as described in earlier studies (Dong et al., 2017; Shao et al., 2018; Shao et al., 2021). *E. coli* strains C41(DE3) and KNabc were used in the HadS production. Electrocompetent *E. coli* cells were prepared and electroporated following established protocols (Dong et al., 2017; Shao et al., 2018; Shao et al., 2021).

**Table 1:** Bacterial strains and plasmids used in this study.

Strains or plasmids	Relevant phenotype or genotype	Source
Strains		
*Halobacillus andaensis* NEAU-ST10-40^T^	Type strain of *H. andaensis*, a moderate halophile	Isolated and identified by our group (Wang et al., 2015)
*Escherichia coli* KNabc	∆*nhaA*∆*nhaB*∆*chaA*	Donated by Professor Terry A. Krulwich (Nozaki et al., 1996)
*E. coli* C41(DE3)	F^−^*ompT gal dcm hsdS*_B_(r_B_^−^ m_B_^−^) (DE3)	Takara Biotechnology (Dalian) Co., Ltd., China
Trans1-T1	Chemically competent cell	TransGen Biotechnology, China
Plasmids		
pUC18	Cloning vector	Takara Biotechnology (Dalian) Co., Ltd., China
pUC-SL40	pUC18 carrying a 2,484-bp DNA fragment with saline–alkaline tolerant gene	This study
pUC-HadS	pUC18 carrying the *hadS* gene with its native promoter	This study
pET19b	Over-expression vector, Amp^R^	Novagen Ltd., USA
pET19b-HadS	pET19b carrying the *hadS* gene fused in frame with an N-terminal His_6_ tag preceded by a T7 promoter	This study
pEASY T3	Cloning vector, Amp^R^	TransGen Biotechnology, China
pEASY T3-truncated YfmM	pEASY T3 carrying C-truncated uup gene with its native promoter	This study

### 2.2 Screening and subcloning of saline-alkaline tolerance-related genes

**Figure 1:**
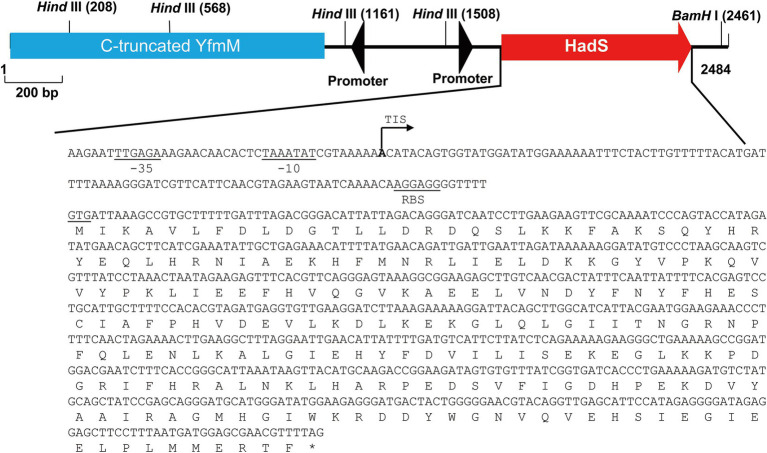
Mapping of the inserted DNA fragment in the recombinant plasmid pUC-SL40. One C-truncated ORF1, designated YfmM (reverse complementary direction), and one intact ORF2, designated HadS, are included in a 2,484-bp DNA fragment (GenBank accession No. PX967208) inserted into the recombinant plasmid pUC-SL40, the latter is preceded by a respective promoter-like sequence and a respective Shine-Dalgarno (SD) sequence. The predicted promoter sequence (−35 region and −10 region), the SD sequence, the ribosomal binding site (RBS), and the initiation codon GTG of HadS are underlined. The transcription initiation site (TIS) is highlighted in bold and also marked with the leftward arrow. The stop codon TAG of ORF2 is indicated by the asterisk.

The *Sau*3AI-partially-digested genomic DNA from *H. andaensis* NEAU-ST10-40^T^, ligated with the BamHI-digested pUC18, was screened for saline–alkaline tolerance-related genes by functional complementation with *E. coli* KNabc on the LBK medium plates containing 0.2 M NaCl (Shao et al., 2018; Shao et al., 2021). This was established by the result that a 2,484-bp DNA fragment was obtained in this study. For the identification of the exact ORF with saline–alkaline tolerance, each ORF included in the 2,484-bp DNA fragment inserted in the recombinant plasmid pUC-SL40 was subcloned separately. The subcloning strategy for HadS gene is shown in Figure 1. The insertion direction of pUC18 was M13F, and a *Hind*III site was present after the 2,484 position. Therefore, the *Hind*III and *BamH*I double enzyme cleavage fragments (2461–1,508 = 953 bp) were recovered and reconnected with the *Hind* III and *BamH* I double enzyme cleavage fragments of pUC18(pUC18-HadS). The full-length *HadS* gen*e* was subcloned from the original recombinant plasmid pUC-SL40 into the expression vector pET19b (Novagen Ltd., USA) in frame with an N-terminal His_6_ tag. A C-terminal truncated *yfmM* gene, preceded by its native promoter and ribosomal binding site (RBS), was subcloned from the original recombinant plasmid pUC-SL40 into the cloning vector pEASY T3 (Transgen Biotech, Beijing, China) (Shao et al., 2018; Shao et al., 2021; Zhang et al., 2020). The sequences of primers used for gene subcloning are listed in Table 2. All resultant constructs, including pUC-HadS, pEASY T3-truncated YfmM, and pET19b-HadS (Table 1), were verified by sequencing analysis.

**Table 2:** Oligonucleotide primers used for subcloning.

Primer ID	Primer sequence (5′ to 3′)
C-truncated YfmM with its native promoter _F	TATGCCTCTCCTTAAACCAT
C-truncated YfmM with its native promoter_R	TTACCACCAAAAGGGCTAGG
HadS_F	CATATGGTGATTAAAGCCGTGC (*Nde*I, underlined)
HadS_R	GGATCCCTAAAACGTTCGCTCC (*BamH*I, underlined)

### 2.3 SDS-PAGE and western blot

Cell extract, membrane fraction (everted membrane vesicles), and cytoplasmic fraction were prepared by the following procedure. *E. coli* KNabc cells carrying pET19b-HadS and the empty vector pET19b (as a negative control) were grown in LBK medium to an OD_600_ of approximately 1.0. The pET19b-HadS exhibited stable basal expression in *E. coli* KNabc without induction, consistent with well-known leaky basal expression of pET-series vectors (Rosano and Ceccarelli, 2014). Cells were harvested by centrifugation at 5,000 × *g*, 4 °C for 10 min. The harvested cells were washed thrice with a buffer containing 10 mM Tris–HCl (pH 7.5), 140 mM choline chloride, 0.5 mM dithiothreitol (DTT), 250 mM sucrose, one protease inhibitor tablet (Roche), and 1 mM phenylmethylsulfonyl fluoride (PMSF). Cell lysis was performed using a JG-1A French pressure cell press (NingBo Scientz Biotechnology Co., Ltd., China) at 2,000 psi. The cell lysate was centrifuged at 5,000 ×, 4 °C for 10 min to remove cell debris and unlysed cells. A small portion of the supernatant was collected as the total cell extract, which contained both membrane and cytoplasmic fractions. The remaining supernatant was further ultracentrifuged at 100,000 × g for 1 h to separate the membrane fraction (pellet) from the cytoplasmic fraction (supernatant). A small portion of the cytoplasmic fraction was also sampled. The membrane fraction was resuspended in the same buffer. Notably, the cytoplasmic membrane prepared by this method exist as inside-out vesicles and are therefore designated everted membrane vesicles. All prepared samples were stored at −80 °C until use.

Localization of HadS in the cell membrane or cytoplasm were determined following established protocols (Shao et al., 2021). The samples representing the cell extract, membrane fraction, and cytoplasmic fraction prepared from *E. coli* KNabc cells carrying pET19b-HadS or the empty vector pET19b (as a negative control) were subjected to sodium dodecyl sulfate-polyacrylamide gel electrophoresis (SDS-PAGE) and Western blotting analysis. The equal amounts of samples were prepared from the same volume of cell lysate, and the loading amount for each sample (equivalent to 50 μg of total membrane protein in membrane fraction) were standardized. The His_6_-tag detection was performed using a mouse anti-His_6_-tag antibody (Beyotime Biotechnology Co., Ltd., China) and a goat anti-mouse secondary antibody conjugated with a horseradish peroxidase (HRP) (Nachuan Biotechnology Co., Ltd., Changchun, China). Luminata crescendo Western HRP substrate (Beyotime Biotechnology Co., Ltd., China) was used, and the chemiluminescence detection was conducted using a Tanon-5200 multi chemiluminescence imaging system (Tanon Co., Ltd., China).

### 2.4 Na^+^/H^+^ antiport assay

Na^+^/H^+^ antiport activity was measured according to the earlier protocol (Shao et al., 2018; Shao et al., 2021). The vesicles (equivalent to 20 μg of total membrane protein) were re-suspended in the assay mixture containing 140 mM choline chloride, 5 mM Mg_2_SO_4_, 1 μM acridine orange at the indicated pH of 6.5–9.5 using 10 mM Bis-Tris propane (BTP) buffer. The fluorescence quenching with the acridine orange as a pH indicator was initiated by the addition of Tris-D-lactic acid (10 mM), which drives the respiration-coupled proton translocation into vesicles. Once fluorescence quenching reached a steady state, a respiration-dependent proton gradient was constructed. After NaCl with high purity (99.9995%, Sigma-Aldrich Co. LLC.) was added to the final concentration of 10 mM, the fluorescence could be dequenched on the basis of Na^+^ influx into the vesicles in exchange for proton efflux. High-purity NaCl was used to eliminate trace sodium impurities present in low-purity salts, which would otherwise introduce undesired background Na^+^, cause non-specific fluorescence dequenching interference, and compromise the accurate quantification of Na^+^/H^+^ antiport activity. The ratio of fluorescence dequenching induced by NaCl to the quenching caused by Tris-D-lactic acid was recorded as a measure of Na^+^/H^+^ antiport activity. Fluorescence was monitored using a Hitachi F-7000 fluorescence spectrophotometer (Hitachi Ltd., Tokyo, Japan) at excitation and emission wavelength of 492 and 526 nm, respectively.

### 2.5 Measurement of intracellular Na^+^ content

Intracellular Na^+^ content was measured according to the previously described protocols (Zhang et al., 2020). The salt-sensitive *E. coli* KNabc mutant was employed in intracellular sodium detection in this study, as this strain reliably reflects changes in sodium accumulation and minimizes non-specific sodium adsorption, thereby ensuring accurate and reliable measurements. Cultures of *E. coli* KNabc transformants with either pUC18-HadS or pUC18 (negative control) were grown overnight, and 1% pre-cultures were inoculated into 200 mL of fresh LBK broth. Cells were harvested at OD_600_ nm of 1.0, washed thrice with a 100 mM Tris–HCl buffer (pH 7.5) containing 140 mM choline chloride and 0.2% glucose, and finally resuspended into 50 mL of the buffer at 4 °C. To initiate the reaction, NaCl was added to the cell suspensions at the final concentration of 0.2 M. The cell suspensions were then incubated at 25 °C for 0–180 min, and 3 mL aliquots were collected in triplicate at designated time points. The samples were centrifuged at 10,000 × *g* for 1 min at 4 °C and washed thrice with the ice-cold buffer to terminate the reaction. Subsequently, the pellets were resuspended in 2.5 mL of 5% trichloroacetic acid (TCA) to lyse the cells. The suspensions were then passed through a 0.22-μm polyethersulfone (PES) membrane to remove debris. Intracellular Na^+^ ion contents were determined using an atomic absorption spectrophotometer iCE-3500 (Thermo Fisher Scientific (China) Co., Ltd).

### 2.6 Fluorescent assay

Fluorescence detection was performed based on the catalytic principle that haloacid dehalogenase can catalyze the dehalogenation of 2-chloropropionic acid (2-CP) to lactic acid, thereby altering the pH of the weakly buffered reaction system (Chen et al., 2024a; Sunder et al., 2024; Dockalova et al., 2020; Aslan-Üzel et al., 2020). *E. coli* C41(DE3) transformants harboring either pET19b-HadS or the empty pET19b vector (negative control) were cultured overnight. Each overnight pre-culture was inoculated at 1% (v/v) into 200 mL of fresh LB broth. When the cultures reached an OD_600_ of 0.6–0.8, protein production was induced with 0.5 mM isopropyl *β*-D-1-thiogalactopyranoside (IPTG), and the cells were further cultured overnight. Subsequently, the cell pellets were washed twice with 20 mM HEPES buffer (pH 8.25), and total membrane fractions were prepared following established protocols (Dong et al., 2017; Shao et al., 2018; Shao et al., 2021). The only difference was that cell extracts were prepared by sonication using an ultrasonic cell disruptor (JY92-11DN; Scientz Biotechnology Co., Ltd., Ningbo, China) instead of high-pressure homogenization. Cell membrane from *E. coli* C41(DE3) transformants harboring pET19b-HadS or pET19b were resuspended in the same buffer. The suspension was supplemented with 2-CP to a final concentration of 30 mM (pH 8.25) and incubated at 30 °C with shaking at 800 rpm for 10–60 min. Subsequently, 10 μM of 8-hydroxypyrene-1,3,6-trisulfonate sodium (HPTS) was added, and the fluorescence spectra were recorded. To determine the pH sensitivity of the fluorescence indicator, 20 mM HEPES buffers at various pH values (ranging from 6.0 to 9.0) were prepared by adjusting with NaOH. Then, 10 μM of HPTS was added, and the fluorescence spectra of these solutions were recorded using a fluorescence spectrophotometer. All fluorescence measurements were performed using a Hitachi F-7000 fluorescence spectrophotometer (Hitachi Ltd., Tokyo, Japan) at excitation and emission wavelength of 375 and 512 nm, respectively.

### 2.7 DNA manipulation and sequence analysis

Preparation of plasmid DNA, extraction of genomic DNA, restriction enzyme digestion, and ligation were performed following previously described protocols (Shao et al., 2018; Shao et al., 2021). DNA sequencing was conducted by the Beijing Genomics Institute (Beijing, China). The open reading frame (ORF) and hydrophobicity analyses were carried out using DNAMAN 8.0 software. Protein sequence alignment was performed through the National Center for Biotechnology Information (NCBI) tool^1^. Promoter prediction was performed using http://www.fruitfly.org/seq_tools/promoter.html, and the transmembrane segment prediction was carried out using https://dtu.biolib.com/DeepTMHMMJeppe (Hallgren et al., 2022). An amino acid sequence logo was created using http://weblogo.threeplusone.com/create.cgi, and the protein structure was predicted using https://alphafoldserver.com/.

### 2.8 Structure modeling of HadS

The amino acid sequence of HadS was submitted to AlphaFold 2 for structure prediction, and the resulting three-dimensional (3D) model was exported in protein data bank (PDB) format. The PDB file was visualized and adjusted using PyMOL, including color scheme optimization and view angle adjustment. The rendered image was further imported into Adobe Illustrator 2025 for manual annotation and final figure preparation.

### 2.9 Nucleotide sequence accession number

The nucleotide sequence reported in this study has been submitted to the GenBank database with the accession number PX967208.

## 3 Results

### 3.1 Cloning and sequence analysis of a saline-alkaline tolerance-related gene

To clone the gene conferring saline-alkaline tolerance, the *Sau*3AI-digested genomic DNA from strain NEAU-ST10-40^T^ was litigated with the cloning vector pUC18 and electroporated into a major Na^+^/H^+^ antiporter-deficient *E. coli* mutant KNabc (∆*nhaA*∆*nhaB*∆*chaA*) (Nozaki et al., 1996). Transformants were screened by functional complementation on the LBK medium plates containing 0.2 M NaCl, as described in earlier studies (Dong et al., 2017; Shao et al., 2018; Shao et al., 2021). As a result, one recombinant plasmid designated pUC-SL40 enabled *E. coli* KNabc cells to grow on the LBK medium plate containing 0.2 M NaCl. Sequence analysis reveale that a 2,484-bp DNA fragment contained one C-truncated ORF1 and one intact ORF2 (Figure 1). ORF1 and ORF2 were annotated as a putative ABC transporter ATP-binding protein (YfmM accession No. GGF13062.1) and a putative L-2-halogenated acid dehalogenase of the HAD superfamily (accession No. GGF13042.1) in the published genome of *H. andaenesis* NEAU-ST10-40^T^ (accession No. BMEL01000001.1), respectively. This study also submitted the amino acid sequence to the GenBank database and re-designated ORF2 (accession No. PX967208.1) as HadS for a HAD superfamily member with saline–alkaline tolerant ability.

### 3.2 Identification of the exact saline-alkaline tolerant gene

**Figure 2:**
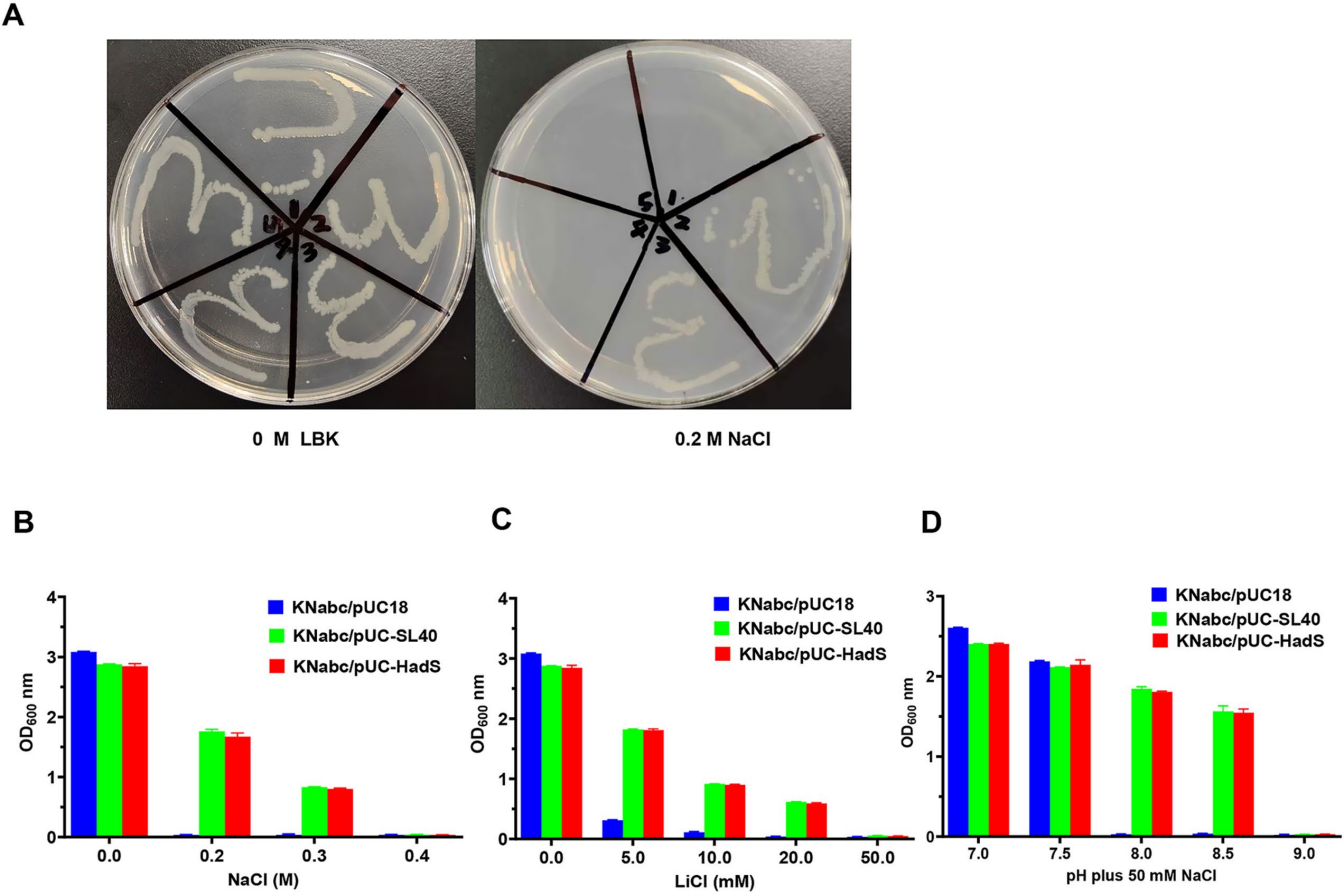
Growth tests for *E. coli* KNabc transformants under saline or alkaline conditions. For the complementation test **(A)**, *E. coli* strains KNabc transformant cells were grown on the LBK medium plates at pH 7.0 either without NaCl or supplemented with 0.2 M NaCl. (1) KNabc/pUC18, (2) KNabc/pUC-SL40, (3) KNabc/pUC-HadS, (4) KNabc/pEASY-T3, and (5) KNabc/pEASY-T3-truncated YfmM. For the growth tests under saline or alkaline conditions, *E. coli* KNabc/pUC-SL40, pUC-HadS, and KNabc/pUC18 were grown in the LBK media containing 0–0.4 M NaCl **(B)** or 0–50 mM LiCl **(C)**, or with **(D)** the addition of 50 mM NaCl at the pH 7.0–9.0. The abovementioned cell growth was ended after 24 h and the values for OD_600_ nm then evaluated. Data are presented as mean ± SD of three independent replicates.

For identification of the gene conferring saline–alkaline tolerance, the *HadS* gene, preceded by its native promoter and ribosomal binding site (RBS) was inserted in the M13F orientation of pUC18, with the subcloning strategy (Figure 1). Specifically, the *Hind* III and *BamH* I double enzyme cleavage fragments (2461–1,508 = 953 bp) were recovered and reconnected with the *Hind* III and *BamH* I double enzyme cleavage fragments of pUC18 (pUC18-HadS). The C-truncated *yfmM* gene, also preceded by its native promoter and RBS, was subcloned from the original recombinant plasmid pUC-SL40 into the cloning vector pEASY T3 (Transgen Biotech, Beijing, China). The resultant constructs were designated pUC18-HadS and pEASY T3-C-truncated YfmM (Table 1). Each subclone was verified by the functional complementation assays using their corresponding transformants in *E. coli* KNabc. In Figure 2A, all the transformants of *E. coli* KNabc grew normally in the absence of NaCl. In contrast, *E. coli* KNabc/pUC18-HadS exhibited growth similar to KNabc/pUC-SL40 in the presence of 0.2 M NaCl, whereas the negative controls KNabc/pUC18 or KNabc/pEASY T3 and KNabc/pEASY T3-C-truncated YfmM showed no growth under the same conditions (Figure 2A). These results indicate that HadS confers saline–alkaline tolerance to *E. coli* KNabc cells.

### 3.3 Resistance of HadS to the salts and alkaline pH

To further test the ability of HadS to induce salt tolerance, *E. coli* KNabc/pUC-SL40, KNabc/pUC-HadS, and KNabc/pUC18 were grown in LBK medium containing 0–0.4 M NaCl or 0–50 mM LiCl. The *E. coli* KNabc/pUC-SL40 and KNabc/pUC-HadS grew in the presence of 0.2–0.3 M NaCl or 5–20 mM LiCl; however, *E. coli* KNabc/pUC18 as a negative control failed to grow in the presence of 0.2 M NaCl or 5 mM LiCl (Figures 2B,C). In order to analyze the resistance of HadS to alkaline pH, *E. coli* KNabc/pUC-SL40, KNabc/pUC-HadS, and KNabc/pUC18 were grown in the LBK medium supplemented with 50 mM NaCl at pH 7.0–9.0. The growth of *E. coli* KNabc/pUC18 was greatly reduced under alkaline conditions, particularly at pH 8.0, compared with that at pH 7.0, whereas the expression of *HadS* conferred *E. coli* KNabc cells the capability to grow under alkaline conditions in the presence of 50 mM NaCl (Figure 2D). To further confirm that the salt-tolerance phenotype is directly attributable to HadS, this study performed additional growth assays using the previously constructed pET19b-HadS (Supplementary Figure S1). The results showed that KNabc/pET19b-HadS grew well at 0.2 M NaCl and grew weakly at 0.3 M NaCl, whereas the control strain KNabc/pET19b failed to grow at 0.2 M NaCl. These results confirmed that the improved salt tolerance is consistently caused by HadS across different expression systems, excluding effects from vector background or other unidentified genes.

### 3.4 Homology alignment and structure modeling of HadS

**Figure 3:**
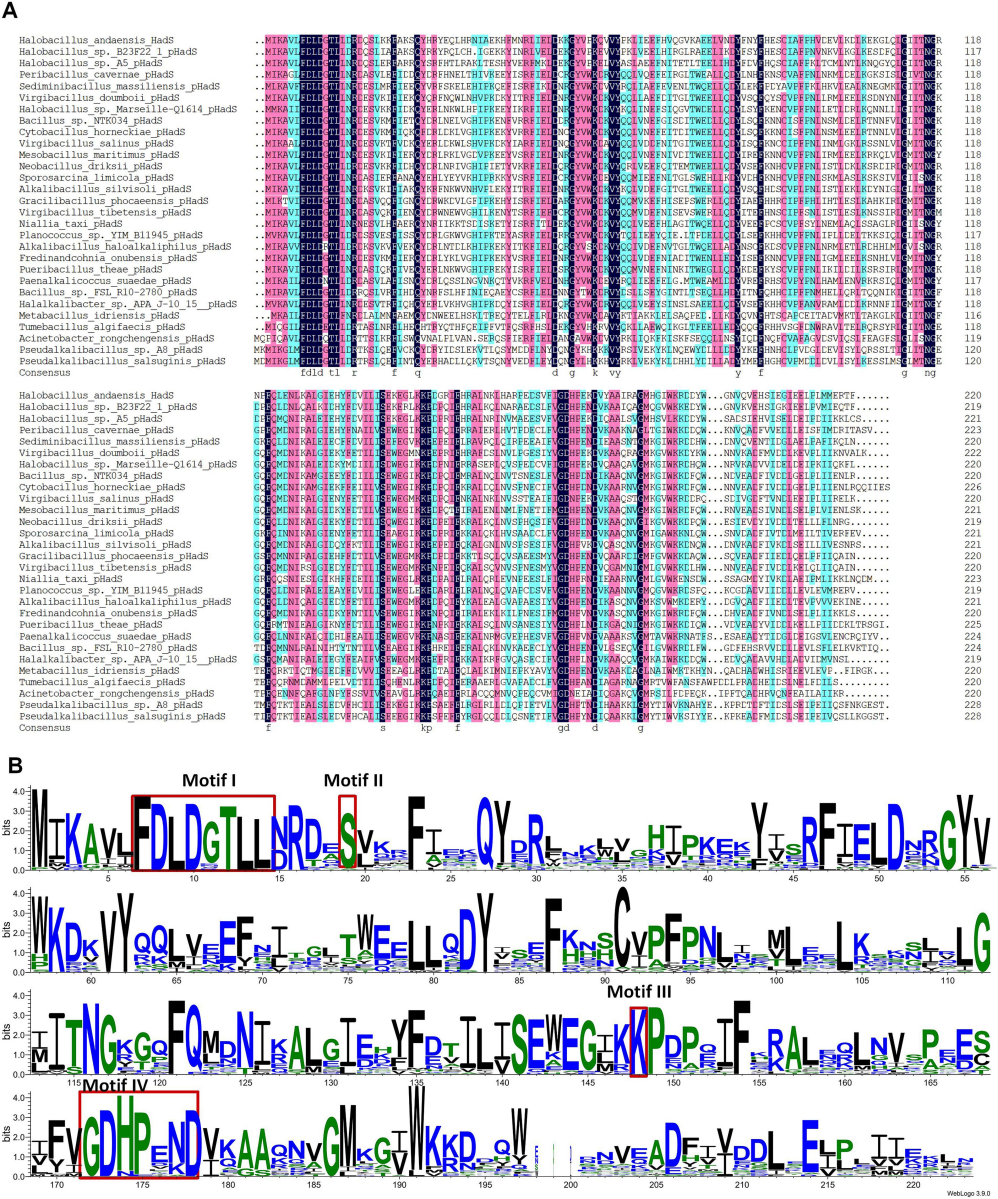
Alignment of HadS with all its selected homologs and its web-based amino acid sequence logo. **(A)** Alignment of HadS with all its selected homologs. HadS was aligned with its selected homologs with 37.16–73.64% identities to show its conserved amino acid residues. Accession version numbers and hosts of the selected homologs are shown in Supplementary Table S1. Shading homology corresponds to 100% (black), ≥75% (pink), ≥50% (light blue), and <50% (white) amino acid identity, respectively. **(B)** HadS sequence weblogo. An amino acid sequence logo was also created by submitting the multiple sequence alignment of HadS with the abovementioned 28 homologs to https://weblogo.threeplusone.com/create.cgi. The heights of amino acid symbols stand for their conservation in the multiple alignment.

**Figure 4:**
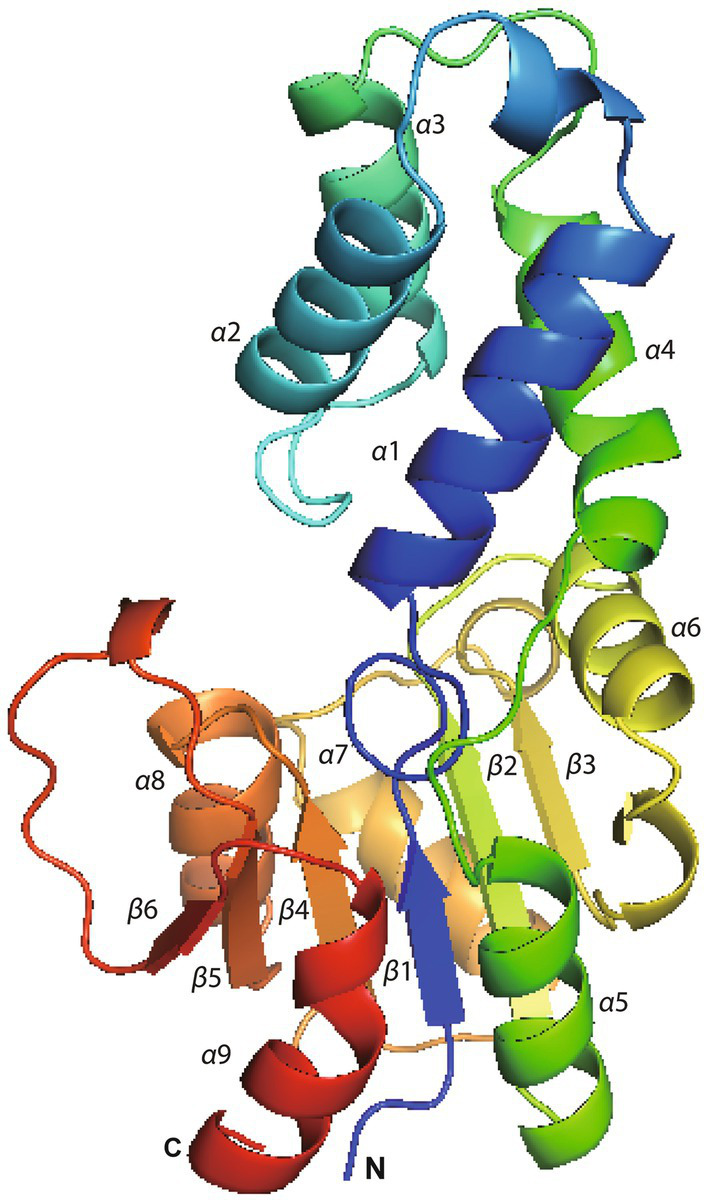
Three-dimensional structure model of HadS. The amino acid sequence of HadS was submitted to AlphaFold2 for structure prediction. The overall structure shows the *α*/*β* hydrolase fold. The model was visualized using PyMOL, with α-helices and parallel β-sheets numbered sequentially.

The homology alignment of the amino acid sequence of HadS is shown in Figure 3A. HadS was aligned with its 28 homologs, and based on multiple sequence alignment, a web-based amino acid sequence logo was generated (Figure 3B) to clearly illustrate residue conservation at positions corresponding to those in HadS within the HAD family proteins. The four highly conserved motifs (Motifs I–IV) were found among the aligned homologs with a wide range of 37.16–73.64% identities (Figure 3B, the red box). As shown in Figure 4, the predicted protein structure contains nine *α*-helices and six *β* -sheets, in which β-sheets of six strands were flanked by α-helices, constituting a Rossmann-like fold characteristic of the HAD superfamily. This structural feature conforms to the typical structural characteristics of 2-halogenated acid dehalogenase. Based on the homology comparison results and protein structure prediction, this study suggests that HadS belongs to HAD family. It should be noted that the conserved Rossmann-like fold and sequence homology cannot sufficiently confirm the definite dehalogenase function, as HAD superfamily includes diverse enzymes with various catalytic activities (Wang et al., 2021).

### 3.5 Hydrophobicity analysis and transmembrane topology prediction

**Figure 5:**
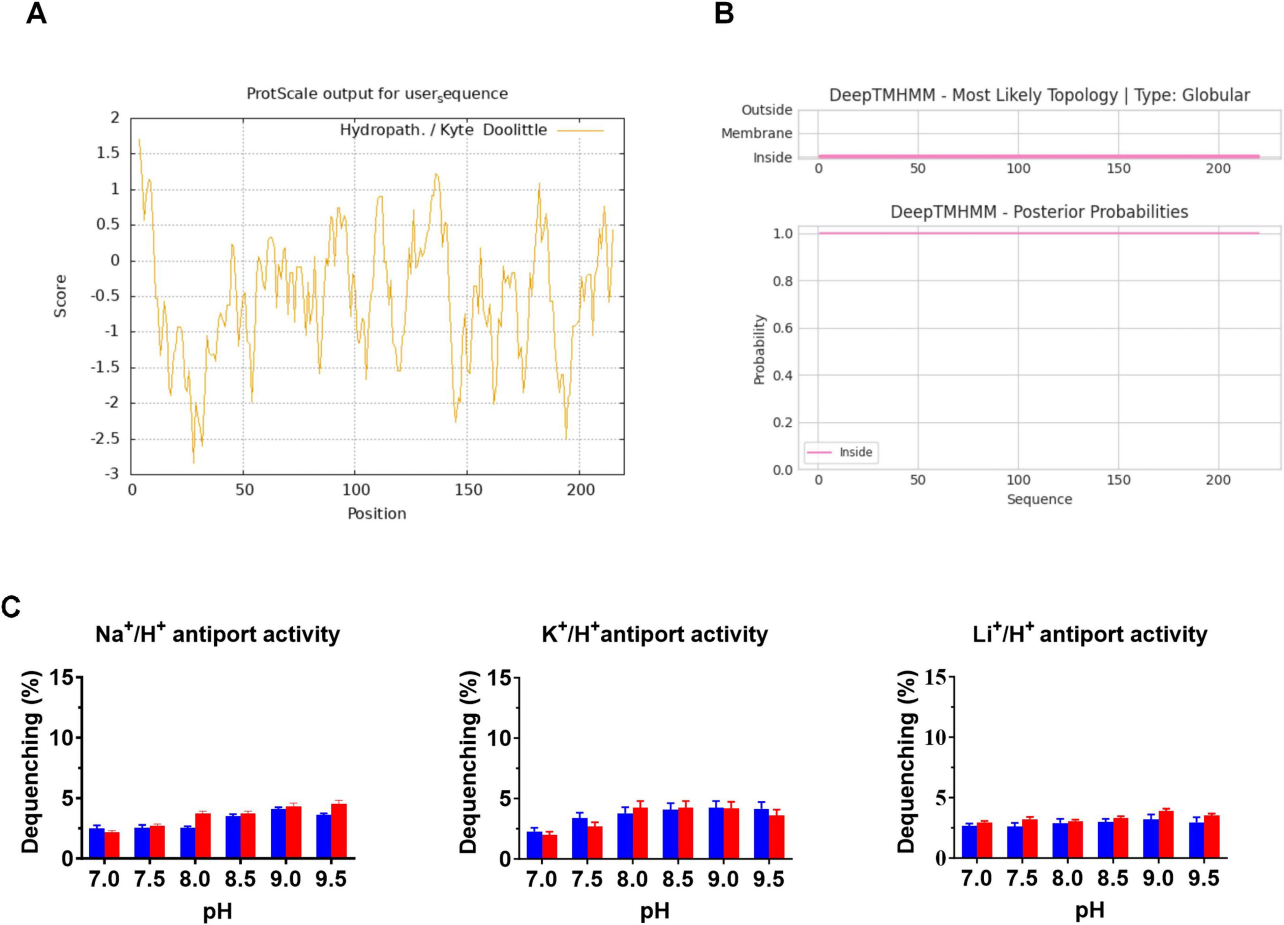
Hydrophobicity analysis, transmembrane topology prediction, and assay for Na^+^(Li^+^, K^+^)/H^+^ antiport activity of HadS. **(A)** Hydropathy patterns of HadS. Hydropathy patterns was created by submitting the sequence of HadS to https://web.expasy.org/protscale/. **(B)** Transmembrane topology prediction of HadS. **(C)** Assay for Na^+^(Li^+^, K^+^)/H^+^ antiport activity of HadS. Na^+^(Li^+^, K^+^)/H^+^ antiport activities were measured in everted membrane vesicles prepared from *E. coli* KNabc transformants with pET19b-HadS (red) or the empty vector pET19b (blue) using the French pressure cell method. Data are presented as mean ± SD of three independent replicates.

The saline–alkaline tolerant genes identified during the previous screening all encode transmembrane proteins with multiple transmembrane domains (Dong et al., 2017; Shao et al., 2018; Shao et al., 2021), and the amino acids that constitute Na^+^/H^+^ transport related proteins have high hydrophobicity, since Na^+^/H^+^ antiporters must be transmembrane proteins of low polarity Wang (Xie et al., 2025; Quinn et al., 2012; Mourin et al., 2019). HadS consists of 220 residues with a calculated molecular weight of 25, 785.6 Dalton and an isoelectric point (pI) of 6.18. Among the 220 residues, 114 residues are hydrophobic. The hydropathy patterns of proteins in HadS is shown in Figure 5A. Transmembrane topology prediction was performed by submitting the amino acid sequence of HadS to https://dtu.biolib.com/DeepTMHMM, and the predicted results indicate that HadS had no transmembrane region (Figure 5B), suggesting that HadS was not a transmembrane protein.

### 3.6 Assay for Na^+^(Li^+^, K^+^)/H^+^ antiport activity of HadS

To test whether HadS functions as a Na^+^(Li^+^, K^+^)/H^+^ antiporter, everted membrane vesicles were prepared from *E. coli* KNabc/pET19b-HadS and KNabc/pET19b (negative control). Na^+^(Li^+^, K^+^)/H^+^ antiport activity was measured by monitoring the dequenching of acridine orange fluorescence upon addition of NaCl, LiCl, or KCl. As shown in Figure 5C, similar Na^+^/H^+^, Li^+^/H^+^ and K^+^/H^+^ antiport activity, with the maximum dequenching of 5%, were detected in everted membrane vesicles from both KNabc/pET19b-HadS and KNabc/pET19b. These results indicate that HadS is not a Na^+^(Li^+^, K^+^)/H^+^ antiporter.

### 3.7 Localization of HadS in the cell by Western blot

**Figure 6:**
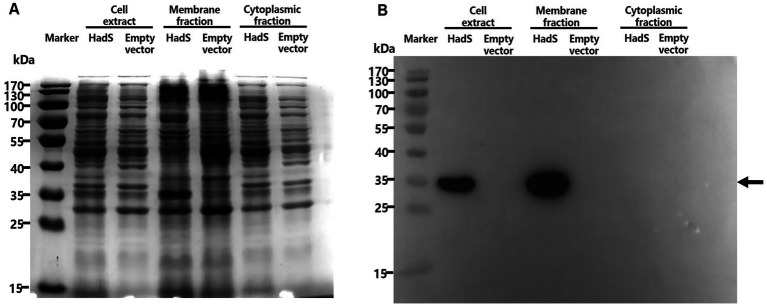
Western blot detection and localization of HadS in *Escherichia coli*. Cell extracts, membrane fractions, and cytoplasmic fractions from *E. coli* KNabc/pET19b-HadS (lanes 1, 3, and 5) and KNabc/pET19b (lanes 2, 4, and 6) were analyzed by SDS-PAGE **(A)** and western blotting **(B)**. The position of target protein HadS fused with a N-terminal His_6_ tag is shown with a solid arrow.

The detection and localization of HadS were carried out using Western blotting analysis. The full-length *HadS* gen*e* was subcloned from the original recombinant plasmid pUC-SL40 in-frame with an N-terminal His_6_ tag into an expression vector pET19b (Novagen Ltd., USA). Therefore, the resultant construct pET19b-HadS, as along with pET19b as a negative control, were selected for the preparation of cell extracts, membrane fractions, and cytoplasmic fractions, followed by the SDS-PAGE (Figure 6A) and Western blotting analysis (Figure 6B). As shown in Figure 6B, strong positive signals for HadS were detected in the membrane fraction and cell extract from the cells of *E. coli* KNabc/pET19b-HadS, but not in KNabc/pET19b. However, no signal was detected in the cytoplasmic fraction from *E. coli* KNabc/pET19b-HadS (Figure 6B). Considering the fact that HadS lacks transmembrane region, it is likely a membrane-associated protein, resulting in the detection in the membrane fraction of *E. coli*. This study found that the heterologous overexpression in *E. coli* may sometimes lead to non-specific membrane association or aggregation. However, the strict exclusive presence of HadS in membrane fractions, coupled with its complete absence in the cytoplasmic fractions, strongly suggests that the observed localization is specific and not an artifact.

### 3.8 Evaluation of the Na^+^ transport function of HadS

**Figure 7:**
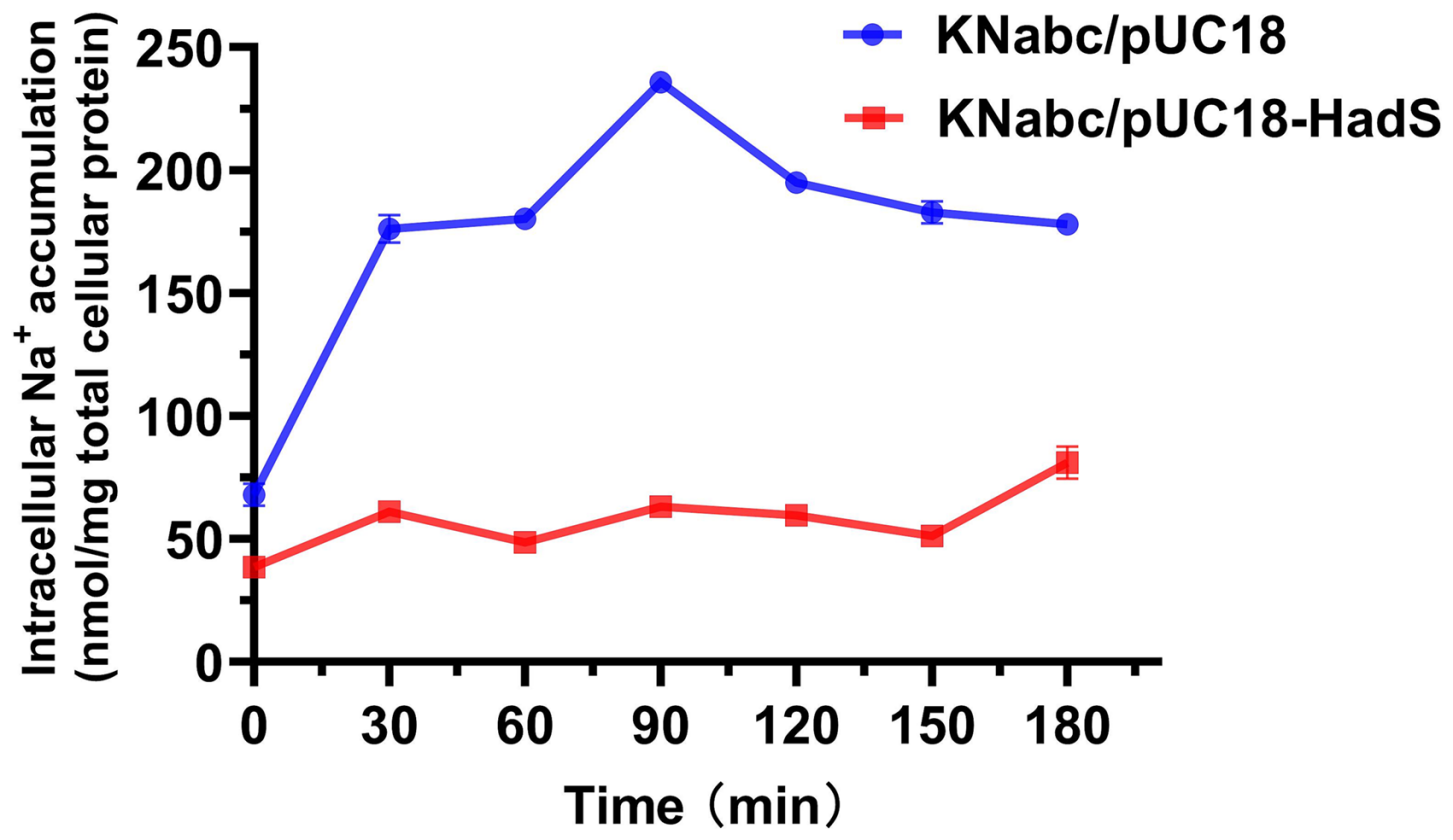
Determination of Na^+^ transport function of HadS using atomic absorption spectroscopy. For the analysis of the intracellular Na^+^ contents, *E. coli* KNabc transformants were grown in Luria–Bertani without the addition of NaCl (LBO), and aliquots of cells were incubated in a 100 mM Tris–HCl buffer (pH 7.5) containing 140 mM choline chloride, 0.2% glucose, and 200 mM NaCl at 25 °C for 0–180 min. Blue filled circle line stands for the growth of KNabc/pUC18 (KNabc/empty vector) and red filled circle line stands for the growth of KNabc/pUC-HadS. Data are presented as mean ± SD of three independent replicates.

For the analysis of the intracellular Na^+^ contents, *E. coli* KNabc transformants expressing HadS or the empty vector were incubated in a 100 mM Tris–HCl buffer (pH 7.5) containing 140 mM choline chloride, 0.2% glucose, and 200 mM NaCl at 25 °C for 0–180 min. As shown in Figure 7, intracellular Na^+^ levels in *E. coli* KNabc expressing pUC18-HadS cells remained relatively stable over time, while *E. coli* KNabc expressing pUC18 cells exhibited a continuous increase in Na^+^ contents prolonged time. Importantly, *E. coli* KNabc cells expressing HadS were found to maintain significantly lower intracellular Na^+^ content than *E. coli* KNabc cells with the empty vector. These results indicate that HadS may extrude excessive Na^+^ from the cells and maintain the lower balance of Na^+^ in the cells.

### 3.9 2-Haloacid dehalogenase activity detection of HadS

**Figure 8:**
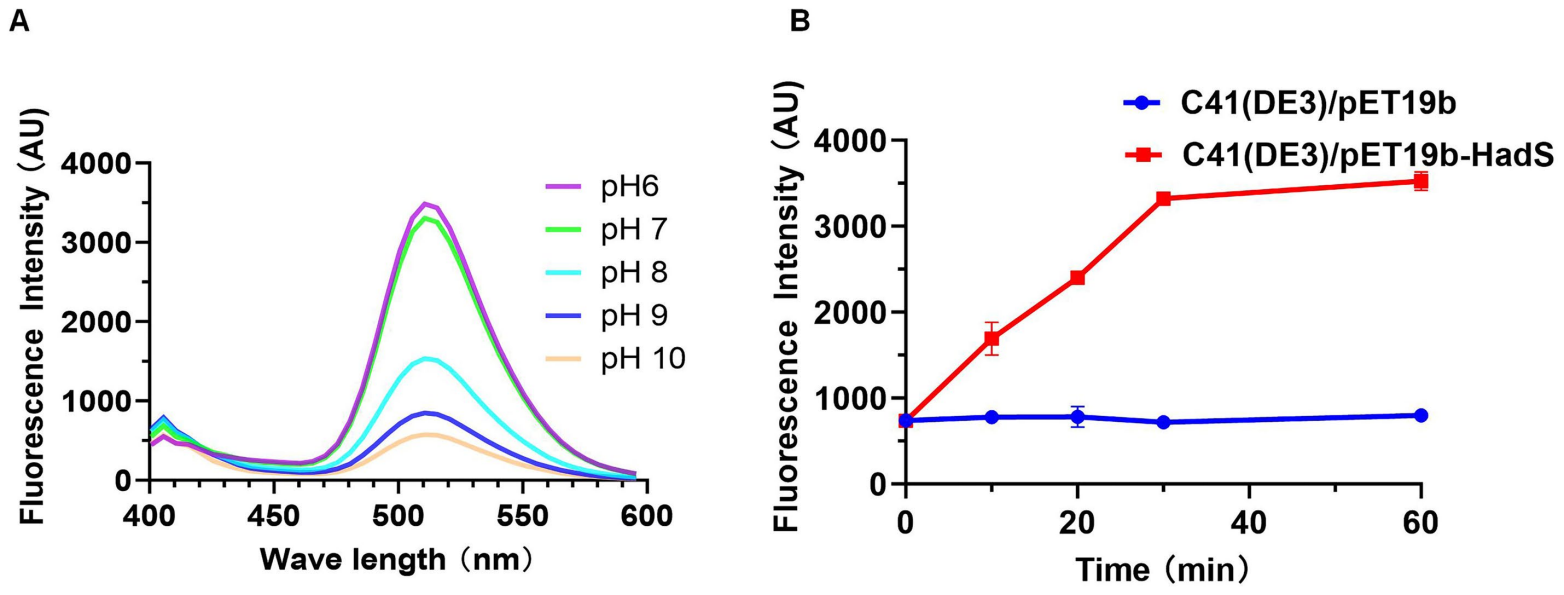
Determination of 2-haloacid dehalogenase activity of HadS using fluorescence spectroscopy method. For the analysis of the 2-haloacid dehalogenase activity of HadS, cell membrane of *E. coli* C41(DE3) transformants with pET19b-HadS or pET19b was resuspended into 2 mL of a 20 mM HEPES Tris–HCl buffer (pH 8.25) with a final concentration of 30 mmol of 2-CP (pH 8.25) and reacted for 10–60 min at 30 °C and 800 rpm, then the addition of 8-Hydroxypyrene-1,3,6-trisulfonate sodium (HPTS) at the final concentration of 10 μM and fluorescence spectrum scanning. All fluorescence measurements were performed using a Hitachi F-7000 fluorescence spectrophotometer (Hitachi Ltd., Tokyo, Japan) at excitation and emission wavelengths of 375 and 512 nm, respectively. Blue filled circle line stands for C41(DE3)/pET19b (empty vector) and red filled circle line stands for C41(DE3)/pET19b-HadS. **(A)** Depicts the pH gradient fluorescence spectra of HPTS and **(B)** depicts the activity of HadS determined by fluorescence spectroscopy. Data are presented as mean ± SD of three independent replicates.

The equilibrium between certain fluorescent molecules and ions varies with pH of the solution, leading to changes in their fluorescence intensity (Sunder et al., 2024). Such molecules may serve as pH indicators. This study selected the fluorescent pH indicator HPTS, which has strong pH sensitivity (Nevolova et al., 2019). The pH gradient fluorescence spectra of HPTS are shown in Figure 8A. When the membrane fraction from cells of *E. coli* C41(DE3)/pET19b-HadS, substrate 2-chloropropionic acid (2-CP), HEPES buffer, and HPTS were all present (HadS group), the fluorescence value of HPTS showed a significant increase (Figure 8B), indicating acidification of the reaction system due to the 2-CP dehalogenation catalyzed by the 2-haloacid dehalogenase HadS. However, no significant change in fluorescence value was detected in the membrane fraction from *E. coli* C41(DE3)/pET19b (Figure 8B). In the dehalogenase activity assay, strict negative control experiments using membrane fractions from the empty vector pET19b strain were performed to exclude non-specific reactions caused by other membrane components. These experiments clearly distinguish the *in vitro* 2-haloacid dehalogenase activity of HadS from its *in vivo* physiological role in saline–alkaline tolerance. The dehalogenase activity detected in this study represents the biochemical catalytic function of the enzyme, while its contribution to salt tolerance reflects the physiological role *in vivo*.

## 4 Discussion

2-Haloacid dehalogenase of the HAD superfamily catalyze the degradation of halogenated compounds that are harmful to the environment, confirming significant applications in the fields of environmental protection and chemical synthesis (Grigorian et al., 2021; Wang et al., 2021). However, few studies have linked HAD superfamily members to saline–alkaline tolerance. This is the first study to report the functional analysis of a HAD superfamily member, designated HadS, from *H. andaensis* NEAU-ST10-40^T^, and the phenotype analysis preliminarily suggest that HadS potentially improves the saline–alkaline tolerance of the host strain. Bioinformatics analysis showed that HadS lacks transmembrane regions, and the fluorescence quenching recovery technology indicated that this protein exhibits no significant Na^+^(Li^+^, K^+^)/H^+^ antiport activity. Therefore, the possibility that HadS functions as a Na^+^(Li^+^, K^+^)/H^+^ antiporter may be ruled out. However, the Western blotting analysis revealed that HadS is located in total membrane-enriched fractions of *E. coli*, suggesting that HadS is likely a membrane-associated protein, although direct structural and biochemical evidence of this unusual localization remains insufficient. In addition, HadS was preliminarily identified to exhibit potential 2-haloacid dehalogenase activity using a fluorescence spectroscopy. In addition, *E. coli* KNabc cells expressing HadS were found to maintain significantly lower intracellular Na^+^ content than *E. coli* KNabc cells with the empty vector, as determined by an atomic absorption spectroscopy method. Based on these experimental results, HadS is preliminarily confirmed as a membrane-associated 2-haloacid dehalogenase. Heterologous expression of HadS may enhance the saline–alkaline tolerance of *E. coli* KNabc while displaying typical 2-haloacid dehalogenase activity. The potential ion translocation and coupling mechanisms during dehalogenation remain to be further explored. Furthermore, this study presents the preliminary evidence that the HAD superfamily member HadS confers saline-alkaline tolerance to *E. coli* KNabc while exhibiting 2-haloacid dehalogenase activity.

Existing researches have clearly demonstrated that 2-haloacid dehalogenases are predominantly present as intracellular soluble proteins (Wang et al., 2021; Zhang et al., 2025; Chen et al., 2024b; Liu et al., 2025; Luo et al., 2025; Edbeib et al., 2020), lacking the typical structural and localization characteristics of membrane-associated proteins. To date, no explicit reports have confirmed the existence of membrane-associated 2-haloacid dehalogenase types. The functional expansion of HadS from canonical dehalogenase activity to a potential role in saline-alkaline adaptation remains speculative and requires further experimental verification. How HadS anchors to the cell membrane to mediate Na^+^ transport function remains unresolved. In particular, this HAD superfamily member HadS appears to have evolved functional versatility, extending its canonical 2-haloacid dehalogenase activity to a potential role in saline-alkaline tolerance. However, given the limited direct experimental evidence presently available, this proposed functional expansion should be interpreted with caution at this stage. This study speculate that such functional versatility may attribute to long-term adaptation of the host under high-salt, high-osmotic environments. In the moderate halophile *H. halophilus,* Cl^−^ ions can increase the activity of glutamine synthetase, thereby increasing glutamate and glutamine concentrations in cells (Saum et al., 2006). The accumulation of glutamine and glutamate helps to maintain intracellular osmotic pressure, serves as a precursor for the synthesis of other compatible solutes (such as proline and betaine), and inhibits oxidative stress. Under Cl^−^ ion deficiency, cell growth is significantly inhibited, while Cl^−^ ion supplementation restores growth (Saum et al., 2006; Mukhtar et al., 2020). The activation of Cl^−^ ion cannot be fully replaced by Br^−^or I^−^ ions, and F^−^ ion even inhibits activity. While Na^+^ ions primarily affects osmotic pressure, Cl^−^plays specific roles in signal transduction and catalytic regulation (Hänelt and Müller, 2013; Xing et al., 2023). Comparative genomic analysis further revealed that the glutamine synthetase (GS) gene in *Halobacillus* species contains a unique Cl^−^-binding domain absent in non-halophilic bacteria. This adaptive evolution may enable rapid response to ion fluctuations in dynamic salinity environments such as salt lakes and tidal flats (Xing et al., 2023; Chen et al., 2020; Gurung et al., 2025). Accordingly, this study tentatively hypothesize that HadS offers 2- haloacid dehalogenase activity to the host *H. andaensis* NEAU-ST10-40^T^, and the released halide ions may potentially promote the accumulation of compatible solutes (such as glutamate and glutamine) in cells, thereby enhancing resistance to high-salt and high-osmotic environments. Nevertheless, direct experimental evidence supporting this hypothesis has not been obtained in the present study. Due to low protein yield and stability in membrane fractions, purified HadS protein was not obtained in the present study. Hence, this study acknowledges this important technical limitation, and further protein purification and detailed functional analysis may be performed in future research. The present results provide preliminary support that HadS is a membrane-associated protein potentially involved in Na^+^ transport, thereby contributing to the maintenance of cellular Na^+^ homeostasis. The subcellular fractionation experiments further confirmed its membrane localization. Future studies may explore the structural and sequential features of HadS to clarify its molecular mechanism, when confirmed. In summary, these findings provide preliminary insights into the functional diversity of HAD superfamily members, highlighting their potential roles in bacterial saline-alkaline tolerance.

## 5 Conclusion

This study provides preliminary evidence for a membrane-associated 2-haloacid dehalogenase, designated HadS, from the moderate halophile *H. andaensis* NEAU-ST10-40^T^. Heterologous expression in *E. coli* KNabc demonstrated that HadS may preliminarily retain catalytic activity against 2-haloacids and confers saline-alkaline tolerance to the host. This discovery challenges the conventional paradigm that 2-haloacid dehalogenases are exclusively soluble proteins, revealing an unexpected structural and functional plasticity within this superfamily. Furthermore, these findings provide novel insights into the regulatory mechanisms by which membrane-associated enzymes confer saline-alkaline stress resistance to the host. These results provide a solid basis for using the membrane-associated 2-haloacid dehalogenase HadS in saline–alkaline bioremediation and constructing stress-tolerant microbial strains.

## Data Availability

The datasets presented in this study can be found in online repositories. The names of the repository/repositories and accession number(s) can be found in the article/Supplementary material.

## Footnotes

1
https://blast.ncbi.nlm.nih.gov/Blast.cgi?PROGRAM=blastp&PAGE_TYPE=BlastSearch&LINK_LOC=blasthome
