# Supplementary material for: HadS, a membrane-associated 2-haloacid dehalogenase from the moderate halophile Halobacillus andaensis NEAU-ST10-40T, enhances saline-alkaline tolerance when heterologously expressed in Escherichia coli KNabc
Source: Front Microbiol. 2026 Jun 8;17:1823317. doi: 10.3389/fmicb.2026.1823317 (PMC13284043; doi:10.3389/fmicb.2026.1823317)
Supplement: Supplementary file 1 [file Supplementary_file_1.docx]

**Supplemental Material**

**HadS, a membrane-associated 2-haloacid dehalogenase from the moderate halophile *Halobacillus andaensis* NEAU-ST10-40^T^, enhances saline-alkaline tolerance when heterologously expressed in *Escherichia coli* KNabc**

Li Shao*, Yunhan Xu , Changjiang He, Shihan Zhao, Yaohua Li, Juquan Jiang*

Department of Microbiology and Biotechnology, College of Life Sciences, Northeast Agricultural University, No. 600 Changjiang Road, Xiangfang District, Harbin, China, 150030

* Correspondence emails: jjqdainty@163.com (J.J.); shao8802022@126.com (L.S.)

**Table S1. Accession.version numbers and hosts of the selected proteins in the protein alignment of Fig. 3**

| **Protein ID** | **Species or strain** | **Protein family or superfamily** | **Identity (%) with HadS** | **Genbank Accesssion No.** |
| --- | --- | --- | --- | --- |
| HadS | *Halobacillus andaensis* | HAD family |  | PX967208.1 |
| pHadS | *Halobacillus sp. B23F22_1* | HAD family | 73.64% | WP_394587219.1 |
| pHadS | *Halobacillus sp. A5* | HAD family | 59.17% | WP_253922902.1 |
| pHadS | *Planococcus sp. YIM B11945* | HAD family | 55.67% | WP_432530993.1 |
| pHadS | *Sediminibacillus massiliensis* | HAD family | 53.21% | WP_077622854.1 |
| pHadS | *Halobacillus sp. Marseille-Q1614* | HAD family | 51.83% | WP_173918919.1 |
| pHadS | *Peribacillus cavernae* | HAD family | 51.38% | WP_126866846.1 |
| pHadS | *Mesobacillus maritimus* | HAD family | 50.69% | WP_404332156.1 |
| pHadS | *Virgibacillus doumboii* | HAD family | 50.68% | WP_164670442.1 |
| pHadS | *Neobacillus driksii* | HAD family | 50.46% | WP_374934856.1 |
| pHadS | *Sporosarcina limicola* | HAD family | 50.00% | WP_192600394.1 |
| pHadS | *Bacillus sp. NTK034* | HAD family | 49.08% | WP_206840136.1 |
| pHadS | *Cytobacillus horneckiae* | HAD family | 48.85% | WP_433959708.1 |
| pHadS | *Fredinandcohnia onubensis* | HAD family | 48.62% | WP_099355132.1 |
| pHadS | *Virgibacillus salinus* | HAD family | 48.61% | WP_092493309.1 |
| pHadS | *Niallia taxi* | HAD family | 47.71% | WP_328037268.1 |
| pHadS | *Gracilibacillus phocaeensis* | HAD family | 47.47% | WP_130859472.1 |
| pHadS | *Alkalibacillus silvisoli* | HAD family | 47.03% | WP_343783168.1 |
| pHadS | *Alkalibacillus haloalkaliphilus* | HAD family | 46.33% | WP_317093200.1 |
| pHadS | *Virgibacillus tibetensis* | HAD family | 46.08% | WP_327609627.1 |
| pHadS | *Pueribacillus theae* | HAD family | 45.28% | WP_116554091.1 |
| pHadS | *Halalkalibacter sp. APA_J-10(15)* | HAD family | 44.44% | WP_247104358.1 |
| pHadS | *Paenalkalicoccus suaedae* | HAD family | 44.09% | WP_176007954.1 |
| pHadS | *Bacillus sp. FSL R10-2780* | HAD family | 43.78% | WP_341037502.1 |
| pHadS | *Tumebacillus algifaecis* | HAD family | 41.40% | WP_094236419.1 |
| pHadS | *Metabacillus idriensis* | HAD family | 41.36% | WP_191566841.1 |
| pHadS | *Acinetobacter rongchengensis* | HAD family | 39.45% | WP_120385091.1 |
| pHadS | *Pseudalkalibacillus salsuginis* | HAD family | 37.38% | WP_236312212.1 |
| pHadS | *Pseudalkalibacillus sp. A8* | HAD family | 37.16% | WP_408010602.1 |

**Fig. S1 Growth of *Escherichia coli* KNabc strains carrying empty pET19b or recombinant pET19b-HadS** **under different NaCl concentrations.**


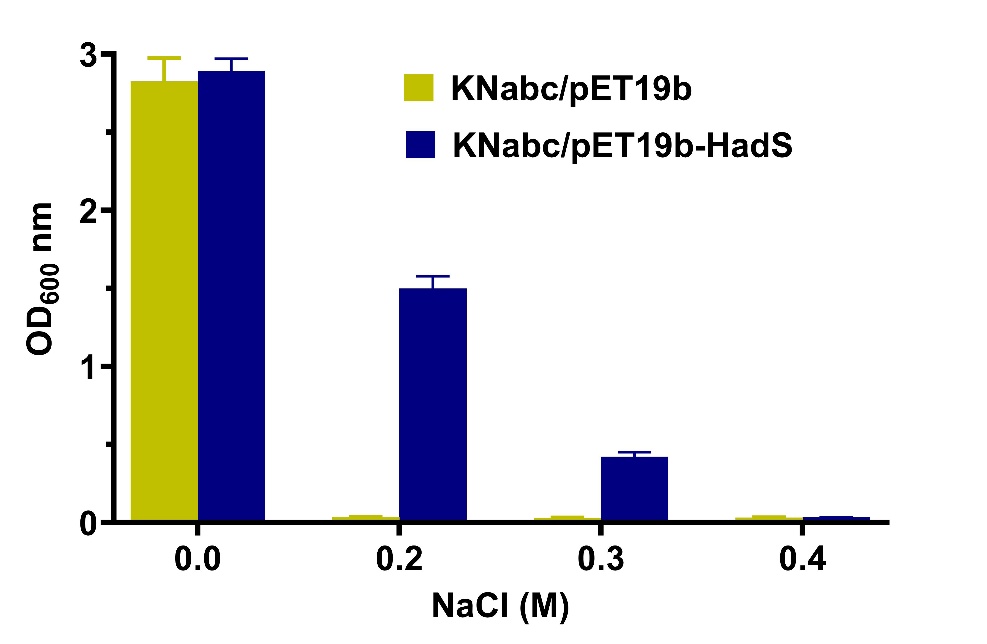
Cells were cultured in 0 M LBK medium supplemented with 0.0, 0.2, 0.3 or 0.4 M NaCl, and growth was determined by measuring the OD₆₀₀ value after 24 h incubation at 37°C. Data are presented as the means ± standard deviations (SD) of three independent experiments.
